# SOS Dental Trauma—An Artificial Intelligence Chatbot via WhatsApp for Guiding Patients After Dental Trauma

**DOI:** 10.1111/edt.70045

**Published:** 2025-12-26

**Authors:** Luana Beatriz das Portas Luiz, Diulia Pereira Bubna, Suzanne Bettega Almeida, Flares Baratto‐Filho, Natanael Henrique Ribeiro Mattos, Manoel Damião Sousa‐Neto, Erika Calvano Küchler, Liliane Roskamp, Cristiano Miranda de Araujo, Bianca Marques de Mattos de Araujo

**Affiliations:** ^1^ School of Dentistry Tuiuti University of Paraná Curitiba Brazil; ^2^ Postgraduate Program in Human Communication Health Tuiuti University of Paraná Curitiba Brazil; ^3^ Department of Dentistry University of Joinville (Univille) Joinville Santa Catarina Brazil; ^4^ Department of Restorative Dentistry, Dental School of Ribeirão Preto University of São Paulo São Paulo Brazil; ^5^ Department of Orthodontics, Medical Faculty University Hospital Bonn Bonn Germany

**Keywords:** artificial intelligence, chatbot, first aid, IADT guidelines, traumatic dental injuries, WhatsApp

## Abstract

**Background:**

Traumatic dental injuries (TDIs) are highly prevalent worldwide and require immediate and appropriate management to ensure favorable outcomes. The aim of the study was to develop and validate SOS Dental Trauma, an interactive artificial intelligence‐based chatbot integrated with WhatsApp. SOS Dental Trauma was designed to provide first‐aid guidance based on the International Association of Dental Traumatology (IADT) recommendations until professional care is available.

**Methods:**

The chatbot was developed using natural language generation techniques, programmed with a structured clinical script strictly limited to dental trauma management in primary and permanent dentitions. The chatbot was implemented with the FastAPI framework in Python, hosted on a cloud server, and integrated into WhatsApp through Twilio. Validation was performed in two stages: (1) assessment by six independent experts in the field of dental traumatology (three general dentists and three endodontists) who evaluated clarity, appropriateness, and coherence of the interaction flow; and (2) performance testing with simulated clinical cases corresponding to trauma types included in the IADT ToothSOS application. Outcomes were defined as accuracy (correspondence to IADT guidelines) and completeness (including all clinically relevant steps). A minimum sample of 384 interactions was calculated, and a performance threshold of 90% accuracy was established. Cases not reaching this threshold were revised and retested.

**Results:**

Experts highlighted the need for more explicit question formulation and adjustments to interaction flow, which were incorporated into the final version. In the initial tests, the chatbot achieved 100% accuracy and completeness for permanent dentition. Nevertheless, it fell below the threshold in cases of primary dentition, particularly luxation and displacement, due to oversimplification of management options. After prompt revisions, the system achieved 100% accuracy and completeness for all trauma types. The chatbot provided structured, accessible, and user‐friendly instructions in alignment with IADT recommendations, demonstrating consistency and reliability.

**Conclusion:**

The SOS Dental Trauma chatbot showed high accuracy and completeness in simulated scenarios, offering immediate, evidence‐based guidance for TDIs in both primary and permanent dentitions. Delivering structured recommendations through a widely used platform such as WhatsApp expands access to reliable first‐aid information, supports patients and caregivers in dental trauma management, and has potential as a complementary tool in dental emergencies and health education.

## 1 Introduction

Traumatic dental injuries (TDIs) refer to any damage involving the teeth, supporting tissues such as the periodontal ligament and alveolar bone, and the soft tissues of the oral cavity. They are generally associated with falls, sports activities, high‐risk interpersonal interactions, or accidents [1, 2]. TDIs represent the second most frequent oral condition and the fifth most prevalent health condition worldwide, with estimates suggesting that at least one billion people have experienced some form of trauma affecting both the primary and permanent dentitions [2, 3]. They are particularly common among children and young adults, accounting for around 5% of all injuries. In permanent teeth, complications may arise, including pulp changes, discoloration, and esthetic, functional, and emotional consequences [2, 4, 5, 6].

As emergencies demand immediate intervention, the general population must be aware of appropriate first aid measures [3]. In clinical practice, the management of TDIs follows the guidelines of the International Association of Dental Traumatology (IADT), which recommends evidence‐based approaches. The accuracy of information and adequate follow‐up are decisive factors for treatment success in primary and permanent teeth. Prognosis, in turn, depends directly on the promptness of the initial intervention. In this respect, providing clear guidance is fundamental to ensure early management, improving treatment adherence, reducing complications, minimizing stress during the accident, and fostering awareness and prevention [7, 8]. In this context, using widely accessible platforms such as WhatsApp becomes a strategic tool to enable the immediate delivery of information in a manner that is both accessible and integrated into patients' daily lives [9].

In recent years, the implementation of artificial intelligence (AI) in healthcare has advanced significantly, driven by the development of large language models that enable applications such as chatbots. These systems are already being used in various health services, aiming to improve efficiency, quality, and care outcomes. Natural language processing (NLP), a branch of AI, seeks to understand and reproduce human language, enabling the accurate interpretation of linguistic data and allowing chatbots to be applied across different medical contexts, from communication and disease detection to diagnostic support and virtual patient assistance [10, 11, 12]. In the specific context of dental trauma, the Tooth SOS application, available for Android and iOS, provides emergency information in accessible language and multiple languages [6]. However, it is a static tool, without AI features [13]. Another relevant initiative is Dental Trauma Evo, an AI‐based chatbot developed to support dentists in the clinical management of dental trauma, offering recommendations based on the IADT guidelines. Nevertheless, this tool is designed exclusively for professional use, focusing on clinical decision‐making support [13]. To date, no AI‐based solutions have been identified that are specifically developed to guide patients on first‐aid measures in cases of dental trauma.

In this context, the aim of the study was to develop SOS Dental Trauma, an interactive AI‐based chatbot integrated with WhatsApp, capable of providing guidance based on the IADT guidelines until face‐to‐face dental care can be delivered.

## 2 Materials and Methods

### 2.1 Chatbot Development

The SOS Dental Trauma was developed to provide immediate guidance, based on the IADT guidelines, on how to proceed in situations of dental trauma prior to professional care. WhatsApp was chosen as the access platform due to its widespread use, allowing instructions to be delivered quickly, easily, and integrated into patients' daily lives.

The system employs natural language generation techniques to organize and draft clinical recommendations clearly and progressively without producing independent responses unrelated to the guidelines. The entire conversational flow was configured to remain strictly limited to dental trauma in both primary and permanent teeth. The chatbot can be accessed directly through the link: https://wa.me/554187947067. In addition, the development code is openly available via Zenodo (DOI: https://doi.org/10.5281/zenodo.17237630).

### 2.2 Architecture and Functional Flow

Interaction with the chatbot occurs through messages sent by the patient via WhatsApp, which are automatically received by an intermediary system (Twilio Sandbox, a tool that integrates WhatsApp with external applications) and forwarded to the chatbot server. This server, implemented in Python (version 3.12) with the FastAPI framework and hosted in a cloud environment (Railway, a platform that enables public deployment of applications), interprets messages according to the conversation history, processes them in line with a set of predefined clinical instructions, and converts them into organized and comprehensible responses.

**FIGURE 1:**
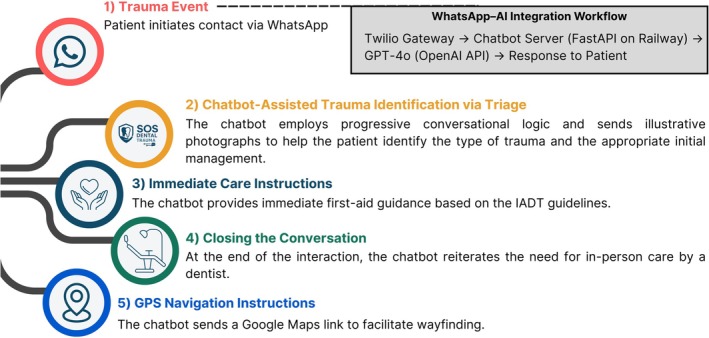
Functional flow of the SOS Dental Trauma chatbot integrated with WhatsApp, based on IADT guidelines.

Linguistic processing was performed using a natural language generation system (OpenAI's GPT‐4o model, configured to operate exclusively within the established clinical guidelines), ensuring that recommendations faithfully adhered to IADT protocols. The history of each conversation was temporarily stored during the interaction to maintain continuity and coherence in the guidance provided. However, it was not retained after the session ended, ensuring information confidentiality. The complete functional flow is illustrated in Figure 1.

### 2.3 Prompt Design and Interaction Logic

The chatbot's behavior was defined through rule‐based prompting, in which a detailed clinical script was programmed to guide all stages of the interaction. This script, prepared by two endodontic specialists with over 10 years of clinical experience and reviewed by a dentist with expertise in dental trauma, strictly follows IADT recommendations.

The logic begins with the system's introduction and identifying the tooth type (primary or permanent), followed by the presentation of different trauma categories, each accompanied by textual descriptions and illustrative images to facilitate recognition by the patient. All figures were created using ChatGPT‐5 (OpenAI, San Francisco, CA, USA), with the specific purpose of illustrating the different types of trauma for each dentition. Based on the trauma selected, the chatbot provides specific immediate management instructions, drafted in accessible language yet technically faithful to the official recommendations. At the end of the interaction, the system closes the conversation in a humanized manner, reiterating the need for in‐person dental care, and may also provide a Google Maps link to help locate nearby healthcare services.

### 2.4 Validation by Specialists

The preliminary version was submitted to evaluation by a panel of six independent experts in the field of dental traumatology, comprising three general dentists and three endodontists, all with over 10 years of clinical experience. These evaluators did not participate in the development of the chatbot. During assessment, they considered the clarity and appropriateness of the recommendations, the suitability of the language used, and the coherence of the interaction flow. The feedback was collectively reviewed and incorporated into the final prompt design and system logic.

### 2.5 Validation Based on IADT Guidelines

The chatbot was tested using simulated clinical cases corresponding to the same types of dental trauma included in the IADT Tooth SOS application, covering both primary and permanent teeth. In each trial, the chatbot was required to provide specific management recommendations for a predefined trauma (e.g., avulsion in a permanent tooth or luxation in a primary tooth). For each scenario, a reference response aligned with the official IADT recommendations was established and used as the gold standard for comparison.

Responses were evaluated in terms of accuracy, defined as the correspondence between the management recommended by the chatbot and that prescribed by IADT, and completeness, defined as including all clinically relevant steps required for the given scenario. A calibrated evaluator conducted testing and analysis. To assess intra‐evaluator consistency, 10% of cases were reassessed after 14 days by the same examiner, and the degree of agreement was estimated using Cohen's kappa coefficient.

Sample size calculation indicated a minimum of 384 interactions for chatbot evaluation, considering an expected accuracy of 50% as a conservative parameter—a value ensuring the most significant possible number of test cases—in addition to a 5% sampling error and a 95% confidence level.

Upon completion of testing, a minimum performance threshold of 90% accuracy was established for each type of trauma assessed. The error was identified whenever this threshold was not achieved; the prompt for the respective trauma was adjusted, and the case was submitted to a new testing round.

## 3 Results

### 3.1 Expert Assessment

The evaluators highlighted the need for greater clarity in the formulation of questions and for adjustments to the interaction flow to facilitate understanding by lay users. These recommendations were incorporated, resulting in responses that were more objective and accessible. Following these modifications, the content was re‐examined and received unanimous approval from the panel of experts.

### 3.2 Performance Assessment

In the initial testing phase, the chatbot performed well in cases involving permanent dentition, with 100% of responses being accurate and complete according to IADT guidelines. By contrast, inconsistencies were observed in trauma cases involving primary dentition, particularly those related to mobility or displacement, in which the accuracy rate remained below the 90% threshold. These shortcomings were associated with the excessive simplification of certain management options, which compromised the completeness of the guidance provided.

**FIGURE 2:**
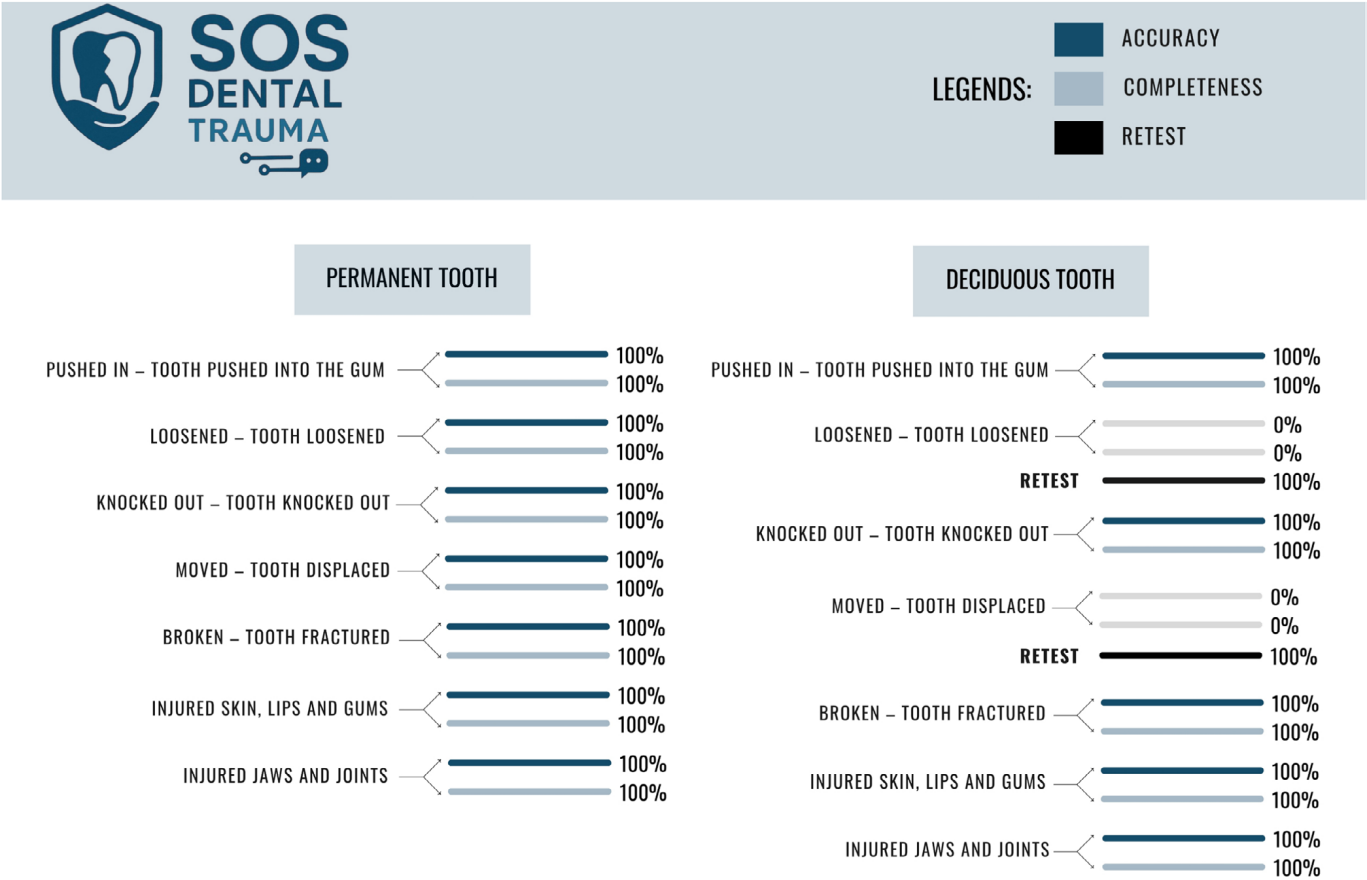
Results of the testing and retesting phases of the responses provided by the SOS Dental Trauma chatbot.

After revising the prompt and correcting the identified inconsistencies, the system showed a significant improvement. In subsequent tests, the responses fully incorporated the recommended management options for primary dentition, achieving 100% accuracy and completeness. The metrics obtained for the different types of trauma are presented in Figure 2.

## 4 Discussion

TDIs are an important public health concern due to their high prevalence and the challenges associated with diagnosis, treatment, and prognosis. Although common, they are often underestimated in clinical practice, which delays appropriate care and increases the risk of social and economic consequences [14, 15]. TDIs affect about one quarter of school‐aged children and roughly one third of adults, most frequently before the age of 19. Limited public knowledge about immediate first‐aid measures reduces the likelihood of favorable outcomes, reinforcing the importance of early intervention and adherence to IADT recommendations [3, 7, 8, 16, 17, 18, 19]. In this context, the present study developed SOS Dental Trauma, an AI‐based chatbot integrated with WhatsApp that provides first‐aid guidance aligned with IADT protocols. The system showed clear, consistent responses and good usability for lay users.

AI has introduced significant advances in dentistry, and chatbots have become increasingly relevant as tools for triage, diagnostic support, and health education. These systems use NLP techniques to understand and generate natural language, enabling dynamic interactions [7, 11, 17, 19]. Among existing resources, the ToothSOS app developed by the IADT offers step‐by‐step first‐aid instructions for different trauma types and provides accessible, illustrated information [7, 20, 21]. However, its availability in a limited number of languages may restrict its reach. SOS Dental Trauma addresses this gap by offering guidance in more than fifty languages and through a conversational format that supports real‐time interaction during emergencies.

Dental Trauma Evo is an AI‐based chatbot designed to support dentists in managing traumatic dental injuries, offering structured, evidence‐based recommendations aligned with IADT guidelines and supporting clinical decision‐making [13]. However, it is restricted to professional use. Çege et al. [3] highlighted that, despite the ability of AI systems to reproduce ToothSOS recommendations, a significant gap persists in providing the public with reliable and understandable first‐aid information. SOS Dental Trauma addresses this gap by offering interactive, real‐time guidance for lay users through a widely accessible platform.

Performance comparisons between ChatGPT‐4o and Gemini Advanced, using ToothSOS as a reference, showed that ChatGPT‐4o performed better in complicated crown fractures, whereas Gemini Advanced outperformed in avulsions. For uncomplicated fractures, luxations, and intrusions, results were similar, although ChatGPT‐4o showed greater variability across evaluations, while Gemini Advanced remained more consistent [3]. These findings reinforce that AI‐based chatbots should complement, rather than replace, clinical judgment. Incorporating IADT guidelines and models tailored to dentistry remains essential for improving reliability [8, 11, 13]. In this study, the ChatGPT‐4o API was integrated into WhatsApp to support user interaction and ensure accessibility in emergency situations.

This study presents some limitations. Firstly, the evaluation was carried out using simulated cases, which, although representative of the most common dental trauma scenarios, may not fully capture the variability and complexity of real‐life situations in real patients. In addition, although the chatbot demonstrated high accuracy and completeness concerning IADT recommendations, its performance in real clinical or community settings has not yet been verified, highlighting the need for future studies to assess its effectiveness and usability under practical emergency conditions. Another aspect to be considered is that the system was designed to provide immediate guidance strictly based on predefined protocols and does not replace professional judgment; therefore, its recommendations should always be regarded as complementary to face‐to‐face dental care.

Despite these limitations, a widely adopted platform such as WhatsApp ensures broad accessibility and population reach, making the system particularly useful in urgent situations outside the clinical environment. Its interactive format supports clear and personalized communication adapted to lay users, thereby promoting greater adherence to appropriate initial measures. Furthermore, integrating official guidelines into simplified language helps reduce management errors, improve prognosis, and minimize complications arising from inappropriate conduct.

In this context, promising prospects emerge for the expansion of the chatbot. Future versions may incorporate multimodal resources, such as the ability for patients to upload images to assist in identifying the trauma or integration with healthcare systems to enable faster referral pathways. The system also has the potential to be incorporated into health education strategies, serving as a training tool for caregivers, teachers, and non‐specialist professionals who frequently encounter TDI in school, sporting, or domestic environments.

## 5 Conclusion

The SOS Dental Trauma chatbot demonstrated good performance, with accurate and comprehensive responses aligned with IADT recommendations for TDI in primary and permanent dentitions. By providing immediate, accessible, and structured instructions through a widely used platform such as WhatsApp, the system can support patients and caregivers in managing dental trauma until professional care is available. Although it does not replace clinical judgment, this AI‐based solution has the potential to broaden access to reliable information.

## Author Contributions

L.B.P.L. contributed to the conception, methodology, investigation, data curation, analysis, drafted, and critically revised the manuscript. D.P.B. contributed to the conception, methodology, investigation, validation, drafted, and critically revised the manuscript. S.B.A. contributed to the investigation, formal analysis, and critically revised the manuscript. F.B.‐F. and M.D.S.‐N. provided supervision, contributed to the data interpretation, and critically revised the manuscript. E.C.K. contributed to the conception, data interpretation, and critically revised the manuscript. L.R. and N.H.R.M. provided supervision, contributed to data interpretation, and critically revised the manuscript. C.M.A. contributed to the conception, design, methodology, software development, data interpretation, visualization, drafted, and critically revised the manuscript. B.M.M.A. contributed to the conception, project administration, methodology, supervision, data interpretation, and critically revised the manuscript. All authors gave their final approval and agreed to be accountable for all aspects of the work.

## Funding

No funding was received for this study.

## Conflicts of Interest

The authors declare no conflicts of interest.

## Data Availability

The source code that supports the findings of this work is openly available on Zenodo (DOI: https://doi.org/10.5281/zenodo.17237630).
